# A Case of Parathyroid Adenocarcinoma and Hyperparathyroidism, When “CRAB” Symptoms Are Not due to a Plasma Cell Myeloma

**DOI:** 10.1155/2020/8815841

**Published:** 2020-08-25

**Authors:** Habib Moshref Razavi, Reza Alaghehbandan

**Affiliations:** ^1^Division of Hematopathology, Department of Pathology and Laboratory Medicine, University of British Columbia, Vancouver, British Columbia, Canada; ^2^Fraser Health Authority, Royal Columbian Hospital, Department of Laboratory Medicine, New Westminster, British Columbia, Canada; ^3^Division of Anatomical Pathology, Department of Pathology and Laboratory Medicine, University of British Columbia, Vancouver, British Columbia, Canada

## Abstract

A previously well 49-year-old patient presented to our hospital with symptomatic hypercalcaemia complaining of polyuria and polydipsia, as well as abdominal and lower back pain (serum/ionized calcium at 3.66 milli mole/l and 1.90 milli moles/l). At admission, he had a normocytic anemia (Hb, 99 g/L) and acute kidney injury (creatinine at 161 *μ*M). His parathyroid hormone (PTH) levels were at 67.6 pico moles/l. A plain X-ray of the lumbar spine showed the presence of a lytic lesion in the L4 vertebrae. CT and MRI confirmed this to be a destructive lesion. A subsequent pan CT scan showed a 2.8 cm complex nodule in the left lobe of the thyroid posteriorly. Excisional biopsy of the resected mass was associated with an infiltrative cellular parathyroid neoplasm with solid and nested architectural growth pattern admixed with hemorrhage and focal calcifications. The tumor showed lymphovascular and perineural invasion. At the time of workup and despite the absence of a positive SPEP/UPEP, a bone marrow biopsy was requested to rule out multiple myeloma. His normocellular bone marrow biopsy showed marked paratrabecular fibrosis and extensive bony remodelling but no metastatic invasion. The diagnosis of a metastatic parathyroid carcinoma was made. He is subsequently considered for palliative radiotherapy to the primary tumor bed and the lumbar spine. In addition, a role for immunotherapy with ipilimumab and nivolumab in context of clinical trials is envisioned and he is being considered for enrollment.

## 1. Introduction

Primary hyperparathyroidism, although usually asymptomatic, can routinely be discovered on biochemical evaluation. One manifestation of primary hyperparathyroidism, parathyroid/hypercalcemic crisis, is defined as calcium levels greater than 3.75 milli moles/l with signs and symptoms of acute calcium intoxication [1]. Parathyroid carcinoma is a rare event [2, 3]. This diagnosis should be considered in patients with primary hyperparathyroidism, who present with a hyperparathroid crisis, or in those who present with a neck mass. Although the classic features of tissue diagnosis include a trabecular pattern, presence of mitotic figures, thick fibrous bands, and capsular and vascular invasion, the two criteria upon which a more definitive diagnosis of parathyroid cancer is reached include local invasion of contiguous structures (such as vascular or neural invasion) and/or invasion of lymph nodes/distant metastases [4–6]. Treatment for this incurable disease includes medical management starting with correction of hypercalcaemia with hydration and bisphosphonates. Cases of refractory hypercalcaemia are treated with calcimimetics such as cinacalcet or the potent inhibitor of bone resorption denosumab [7, 8]. The mainstay of therapy however includes surgical resection including palliative debulking of metastatic disease. In cases of widespread metastatic disease not amenable to surgery, chemotherapy and adjuvant palliative radiation of bone metastases or locoregional disease play a role. However, patients in this stage generally have many comorbidities and an overall poor prognosis [2]. Here, the case of a patient with hypercalcaemia, renal failure, anemia, and lytic lesions (CRAB) is described. This constellation of these symptoms evoked the diagnosis of plasma cell myeloma, which was not diagnosed in the described case.

## 2. Case Report

**Figure 1:**
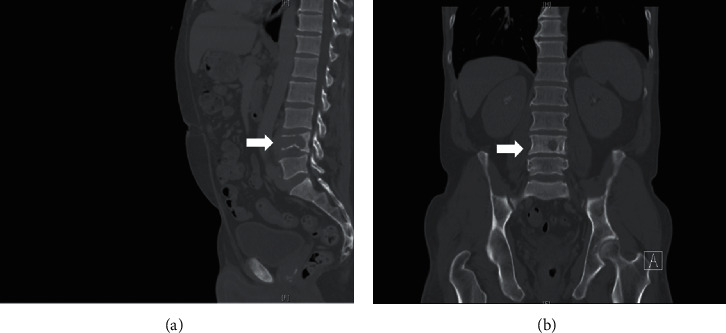
Lumbar CT, left sagittal (a) and coronal (b) planes. A destructive lesion causing pathological fracture of the L4 vertebral body with mild adjacent soft tissue thickening. No abnormal soft tissue in the spinal canal was identified.

**Figure 2:**
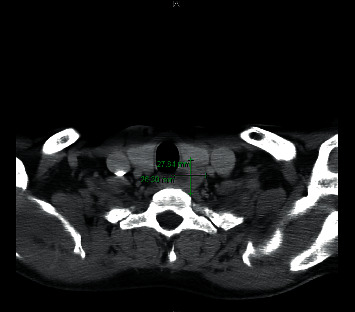
CT neck, transverse plane. A 2.8 × 2.6 cm complex nodule in the left lobe of the thyroid. On further examination by way of a thyroid ultrasound, the lesion was further characterized and described as an echo poor mass showing lobulated margins and coarse internal calcification, highly suspicious for malignancy.

**Figure 3:**
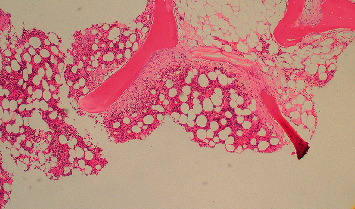
Bone marrow trephine biopsy, H&E × 10. Bony trabecular remodelling and paratrabecular fibrosis are present. The bone marrow biopsy is otherwise normocellular and shows trilinear hematopoiesis.

**Figure 4:**
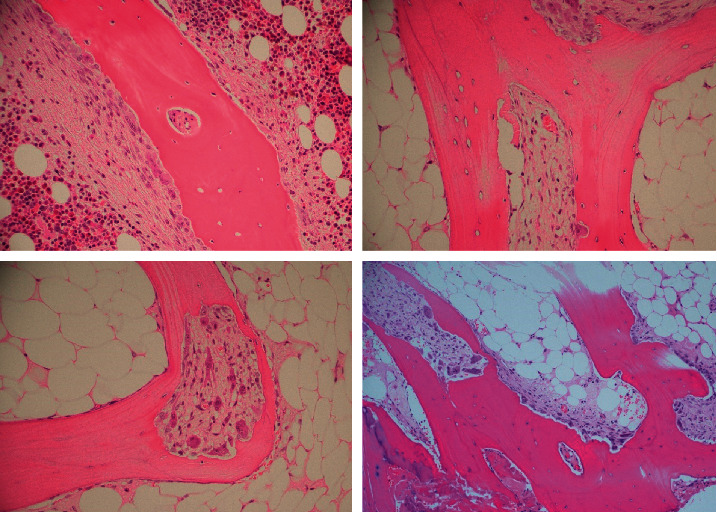
Enlarging Howship lacunae populated by osteoclasts. These enlarge and transect the bone marrow trabeculae forming swaths of intertrabecular fibrosis (lower row, right panel).

**Figure 5:**
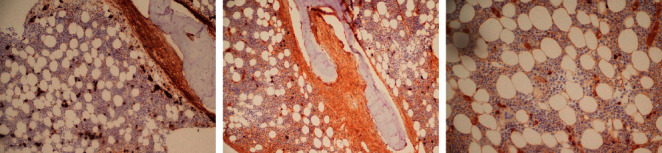
CD 138 and kappa and lambda stains show presence of polytypic plasma cells.

The case of a 49-year-old patient with several years of lower back pain with acute exacerbation is described. His past medical history is notable for epilepsy associated with childhood head trauma. Following a prior left frontal lobe resection of brain scar tissue in 1996, this condition has since resolved. He has a 45 pack-year history of smoking, and until October of 2019, he had been a heavy drinker, consuming up to 15 drinks per day. He works on a boat yard. He reported a 40 pounds weight loss over the previous couple of years. His family history is significant for maternal history of lung cancer. His father had skin cancer. On admission to our hospital, the patient reported some constipation, polyuria, and polydipsia. He denied any flushing, diaphoresis, or presence of renal stones. A routine CBC and chemistry panel showed a normocytic anemia (99 g/l) and much elevated calcium levels (serum/ionized calcium at 3.66 milli moles/l and 1.90 milli moles/l). He also had an acute kidney injury (creatinine at 161 *μ*moles/l). His serum phosphorous was low at 0.6 mmol/l. His 24-hour urine calcium level was increased at 13 mmol/day. His PTH was markedly elevated at 67.6 pico moles/l. Given his lower back pain and CRAB features, a spine X-ray as well as a lumbar CT were done, which showed lytic lesions in the L4 vertebral body (Figure 1). The biochemical correlate, his bone-specific alkaline phosphatase, was accordingly elevated at 170 U/l. His physical examination showed normal vital signs and no physical evidence of lymphadenopathy or splenomegaly. However, palpation of the thyroid revealed a 2 cm nodule on the left lobe. A pan CT showed a 2.8 cm × 2.6 cm complex left lobe thyroid nodule concerning for malignancy (Figure 2). The CT scan also confirmed lytic lesions at L4, as well as more lesions in C6-C7 and 9th ribs. A parathyroid scan done showed increased uptake at the mid and inferior aspect of the left thyroid bed posteriorly. A renal ultrasound showed mild echogenic changes bilaterally, but no hydronephrosis. A fine needle aspiration biopsy of the thyroid mass showed the presence of follicular-like epithelial clusters arranged in papillary and microfollicular architecture. The aspirated cells were stained for synaptophysin and chromogranin and were negative for TTF1 and thyroglobulin immunostains which in context of clinical findings suggested the presence of cellular parathyroid tissue. A parathyroidectomy was attempted. Upon exposure of the thyroid gland, a fibrous tumor with obvious macroscopic invasion of the esophagus, trachea, and left lobe of the thyroid gland was identified and resected. Tumor histology showed an infiltrative cellular parathyroid neoplasm with solid and nested architectural growth pattern admixed with hemorrhage and focal calcifications. The tumor also showed evidence of lymphovascular and perineural invasions consistent with features of a parathyroid carcinoma. In light of CRAB symptoms and in order to rule out a plasma cell myeloma and/or any other bone marrow intrinsic etiology, a bone marrow biopsy was requested. His normocellular bone marrow biopsy showed marked paratrabecular fibrosis (Figure 3). Increased osteoclastic activity showed surface bone excavation and trabecular tunneling. This stemmed from enlarging Howship lacunae, which effectively bisected the trabeculae (Figure 4). CD34 immunoperoxidase for blasts and CD3 (pan T) and CD20 (B) immunoperoxidase stains for T and B lymphocytes respectively were noncontributory. In addition, CD138, kappa and lambda stains showed polytypic plasma cells (Figure 5). Upon diagnosis, the patient was managed with fluids and cinacalcet as well as a one-time dose of bisphosphonates (pamidronate disodium 60 mg I.V.). He was a candidate for palliative radiation to his L4 lesion. There was also a consideration for enrollment in a phase II trial of immunotherapeutic agents ipilimumab and nivolumab which is not available in Canada. This trial is ongoing in the United States. However, unfortunately, the patient has as of this writing been unable to enroll due to traveling restrictions during the worldwide COVID-19 pandemic.

## 3. Discussion

Parathyroid carcinoma is a rare disease. In >90% of cases, it manifests with hyperparathyroidism [9]. The consequences of hyperparathyroidism reflects its physiological role of calcium metabolism including calcium resorption from the bone by way of PTHR1 receptor as well as renal activation of vitamin D and increased intestinal calcium uptake [10]. Pathophysiological consequences of overt hyperparathyroidism may present with renal, musculoskeletal, and neurological abnormalities such as nephrolithiasis/nephrocalcinosis, bone pain, and osteopenia, as well as anxiety and depression. Rarely, patients with parathyroid carcinoma present with clinical outcomes of life-threatening hypercalcaemia including renal failure, cardiac arrhythmias, and coma [11, 12]. Diagnosis requires a high index of suspicion. However, presence of some risk factors is informative. These include albumin-corrected hypercalcaemia (>3 milli moles/l), PTH levels being more than 3 times the upper limit of normal, presence of a neck mass at more than 3 cm along with neck pain and hoarseness as well as imaging evidence of infiltration and/or calcification on a neck ultrasound [13]. Accurate diagnosis is paramount for treatment regimens as pathological correlates such as parathyroid adenoma and hyperplasia exist and have markedly different clinical outcomes [14]. Features when present that are diagnostic of parathyroid carcinoma include unequivocal vascular invasion by the tumor, perineural invasion, gross invasion into adjacent anatomic structures, and distant metastases [15, 16]. Worrisome but nondiagnostic features include the presence of coagulative necrosis, increased mitotic activity (>5/50 high power field), nuclear atypia, presence of macronucleoli, and presence of broad fibrous bands. In most cases, diagnosis is reached by morphology with clinical correlation. However, a panel of biomarkers such as galectin-3, parafibromin, p27, Rb, APC, Bcl-2a, p53, and MIB-1/Ki67 proliferation index may be helpful in borderline cases [17].

Bony and skeletal changes have been attributed to primary and secondary hyperparathyroidism. In fact, bony remodelling due to renal osteodystrophy and/or hyperparathyroidism secondary to intestinal malabsorption and/or gastric bypass surgery is indistinguishable to changes attributed to primary hyperparathyroidism. Enhanced activation of both osteoclasts and osteoblasts results in bone resorption by way of enlarging Howship lacunae, trabecular transection, and paratrabecular fibrosis. Larger lesions may cause the presence of fibrosis in an entire intertrabecular space [18, 19]. Therefore, at least in part, afflicted patients present with a hypoproliferative trilinear hematopoiesis manifesting as cytopenias. Surgical resection remains the mainstay of therapy and if by biopsy a parathyroid carcinoma is diagnosed, further revision surgery may be required for optimal disease control.

In conclusion, here we present a case of a patient with hypercalcaemia, renal dysfunction, anemia, and bony lytic lesions, initially evoking the diagnosis of plasma cell myeloma. However, a bone marrow biopsy as part of an extensive workup showed bone marrow manifestation of primary hyperparathyroidism associated with a hypoproliferative hematopoiesis. While the prognosis of metastatic parathyroid carcinoma remains poor, enhanced understanding of the disease mechanism has provided possible molecular targets such as cyclin D1, beta catenin, and calcium sensing receptors (CaSR) which may offer better outcomes.

## Data Availability

All relevant data have been included within the article.

## References

[1] Lew J. I., Solorzano C. C., Irvin G. L. (2006). Long-term results of parathyroidectomy for hypercalcemic crisis. *Archives of Surgery*.

[2] Wei C. H., Harari A. (2012). Parathyroid carcinoma: update and guidelines for management. *Current Treatment Options in Oncology*.

[3] Ozolins A., Narbuts Z., Vanags A. (2016). Evaluation of malignant parathyroid tumours in two European cohorts of patients with sporadic primary hyperparathyroidism. *Langenbeck’s Archives of Surgery*.

[4] Schantz A., Castleman B. (1973). Parathyroid carcinoma. A study of 70 cases. *Cancer*.

[5] Bondeson L., Sandelin K., Grimelius L. (1993). Histopathological variables and DNA cytometry in parathyroid carcinoma. *The American Journal of Surgical Pathology*.

[6] Quinn C. E., Healy J., Lebastchi A. H. (2015). Modern experience with aggressive parathyroid tumors in a high-volume New England referral center. *Journal of the American College of Surgeons*.

[7] Collins M. T., Skarulis M. C., Bilezikian J. P. (1998). Treatment of hypercalcemia secondary to parathyroid carcinoma with a novel calcimimetic agent. *The Journal of Clinical Endocrinology & Metabolism*.

[8] Karuppiah D., Thanabalasingham G., Shine B. (2014). Refractory hypercalcaemia secondary to parathyroid carcinoma: response to high-dose denosumab. *The European Journal of Endocrinology*.

[9] Fraser W. D. (2009). Hyperparathyroidism. *The Lancet*.

[10] Hannan F. M., Kallay E., Chang W. (2018). The calcium-sensing receptor in physiology and in calcitropic and noncalcitropic diseases. *Nature Reviews Endocrinology*.

[11] Al-Kurd A., Mekel M., Mazeh H. (2014). Parathyroid carcinoma. *Surgical Oncology*.

[12] Kassahun W. T., Jonas S. (2011). Focus on parathyroid carcinoma. *International Journal of Surgery*.

[13] Duan K., Mete O. (2015). Parathyroid carcinoma: diagnosis and clinical implications. *Turkish Journal of Pathology*.

[14] Erovic B. M., Goldstein D. P., Kim D. (2013). Parathyroid cancer: outcome analysis of 16 patients treated at the princess Margaret hospital. *Head & Neck*.

[15] Johnson S. J. (2010). Changing clinicopathological practice in parathyroid disease. *Histopathology*.

[16] Mete O., Asa S. L. (2011). Pathological definition and clinical significance of vascular invasion in thyroid carcinomas of follicular epithelial derivation. *Modern Pathology*.

[17] Shifrin A., Li Volsi V., Shifrin-Douglas S. (2015). Primary and metastatic parathyroid malignancies: a rare or underdiagnosed condition?. *The Journal of Clinical Endocrinology & Metabolism*.

[18] Broadus A. E. (1989). Primary hyperparathyroidism. *Journal of Urology*.

[19] DeVita M. V., Rasenas L. L., Bansal M. (1993). Assessment of renal osteodystrophy in hemodialysis patients. *Medicine*.

